# Ubiquitous Monitoring of Electrical Household Appliances

**DOI:** 10.3390/s121115159

**Published:** 2012-11-07

**Authors:** Jaime Lloret, Elsa Macías, Alvaro Suárez, Raquel Lacuesta

**Affiliations:** 1 Instituto de Investigación para la Gestión Integrada de Zonas Costeras (IGIC), Universidad Politécnica de Valencia, Camino Vera s/n, 46022, Valencia, Spain; 2 Grupo de Arquitectura y Concurrencia (GAC), Departamento de Ingeniería Telemática-Universidad de Las Palmas de Gran Canaria, Campus Universitario de Tafira, Edificios de Telecomunicación, 35017, Las Palmas de Gran Canaria (Gran Canaria), Spain; E-Mails: emacias@dit.ulpgc.es (E.M.); asuarez@dit.ulpgc.es (A.S.); 3 Departamento de Informática e Ingeniería de Sistemas, Universidad de Zaragoza, Ciudad Escolar s/n 44003 Teruel, Spain; E-Mail: lacuesta@unizar.es

**Keywords:** ubiquitous monitor, Internet of Things, electrical household appliances, social networks

## Abstract

The number of appliances at home is increasing continuously, mainly because they make our lives easier. Currently, technology is integrated in all objects of our daily life. TCP/IP let us monitor our home in real time and check ubiquitously if something is happening at home. Bearing in mind this idea, we have developed a low-cost system, which can be used in any type of electrical household appliance that takes information from the appliance and posts the information to the Twitter Social network. Several sensors placed in the household appliances gather the sensed data and send them wired or wirelessly, depending on the case, using small and cheap devices to a gateway located in the home. This gateway takes decisions, based on the received data, and sends notifications to Twitter. We have developed a software application that takes the values and decides when to issue an alarm to the registered users (Twitter friends of our smart home). The performance of our system has been measured taking into account the home network (using IEEE 802.3u and IEEE 802.11g) and the data publishing in Twitter. As a result, we have generated an original product and service for any electrical household appliance, regardless of the model and manufacturer, that helps home users improve their quality of life. The paper also shows that there is no system with the same innovative features like the ones presented in this paper.

## 1. Introduction

Domotics systems traditionally have allowed the adoption of Information and Communication technologies at home. Domotics tries to ensure a higher user comfort, improve security, make energy resource management more efficient and improve communication between the home and users. External access of housing data, appliances, and other facilities will allow greater control and increase the quality of life of people.

The increase of computational capacity and the inclusion of networking technologies in electrical household appliances are revolutionizing the way we interact with our homes. This trend is growing fast and it is opening a number of technological challenges. Moreover, the availability of low cost computational elements (ranging from mobile phone hardware for a few dollars to smart dust technology) is creating a wide spectrum of opportunities for industry and research activities [1]. From the point of view of distributed systems, there is a need to design architectures for enhancing the comfort and safety at home, which deals with issues of heterogeneity, scalability and openness. The development of intelligent sensing agents and sensor-based information appliances will spread pervasive and ubiquitous technology to a multitude of housework tasks. Some studies deal with international selection of leading smart home projects, as well as the associated technologies of wearable/implantable monitoring systems and assistive robotics. For example, in [2] its authors study modern sensor-embedded houses, or smart houses, that not only assist people with reduced physical functions but they help resolve the social isolation issues they may face. The concept of the smart home is a promising and cost-effective way to improve home care for the elderly and disabled people in a non-obtrusive way. This leads to the decentralization of healthcare from the hospital to the home allowing greater independence, while maintaining good health and preventing social isolation. Devices in smart homes operate in a network connected to a remote unit for data collection and processing. Generally, people have different needs and provision from this decentralized system has to be tailored to each individual [1].

The market for networked home appliances and residential gateways is expanding and arouses both research and industry interest. For example *The Smart Kitchen project* [3] evaluates different fieldbus systems with respect to upcoming domotics applications. A number of small, inexpensive devices are networked to implement functions for increasing comfort, security, safety and better energy management at home. Fieldbus systems to Internet gateways are used to access data from the home. They present an example that uses a web-server, which publishes energy measurements on kitchen devices. Reference [3] shows how many small devices can be networked over a fieldbus in a domotics application.

In this environment, household appliances and other devices could be managed independently or collectively. The user must be able to interact with each element or with a group of them. If we focus on standards and existing systems we will check that most of these systems are proprietary. If we desire a domotics system at home we will use these systems several times: Friend, GIV, Biodom, Hometronic, Cardio, Maior-Domo, Concelac, PLC, Dialoc, PlusControl, Dialogue, Simon VIS Domaike, Simon Vox Domolon, Starbox, DomoScope, Vantage, Domotel, VivimatPlus. Other standards, such as x-10, are open. Nevertheless, Pico Electronics LTd circuit is required to use such standard. There are other standards such as Building Automation and Control Network (BACnet), Batiment Intelligent Bus (Batibus), Consumer Electronic Bus (CEBu), European Home System (EHS), European Installation Bus (EIB), Home Bus System (HBS), Home Electronic System (HES), Konnex, and LonWorks that we could use to develop our system. For example, electrical appliances of the home network can be monitored via the Internet using standards such the ZigBee system [4]. Other proposals, like the ones presented in [5], use the existing home infrastructure based also on open industry standards. This system tries to integrate the home network with external networks to easily manage home devices, both locally and remotely. Moreover, new strategies can be applied in these access networks to provide real-time communications [6].

The advent of pervasive computing marked an urgent need for a new generation of intelligent sensing agents and information appliances. It also demanded environments for resource management of broad applications involving loosely coupled, event-driven and diverse information appliances. Internet of Things (IoT) appeared to provide connectivity of any device (thing) to the Internet. Examples of things are: fridges, mobile telephones, food and plants. In IoT things can communicate among themselves or they can communicate with people: new forms of communication are established between people and things and between things. Using wireless connectivity communication can be done anytime, anywhere, for anyone and for anything. IoT also allows the integration of several technologies and communications solutions and it enables communications with and among smart objects [7]. To solve this situation some architectures have been proposed such as the EPC Global Internet of Things and the Ubiquitous ID complex networking system [8]. Key technologies of IoT are RFID technology, sensor network and detection technology, intelligent technology and nano-technology. Some current solutions are being already sold in the market; an example is Samsung Snowing, RF4289 and Side by Side RSG309 models with capabilities such as time control of stored food for notice prior to its expiration date, or the ability to play music online [9]. In all these systems a controller is responsible of requesting information from each appliance.

The number of appliances at home is increasing due mainly to the facilities they offer. Technology is integrated in all that surrounds us, in all objects of our daily life. Household items such as washing machines are improving their performance. Nowadays, washing machines are able to select the type of program, water and temperature according to the washing weight or clothes materials. Another example is the microwave that calculates the elements weight to carry out its tasks.

Monitoring our home in real time (from any location) will let us check if something is happening. We think that a smart house must have appliances (things) able to connect to Internet to notify alarms. These appliances must be wired or wirelessly connected among them and also they must be connected to Internet, for example to tweet alarm messages in the Twitter Social network.

In this research we demonstrate a low-cost communication architecture that increases the safety and quality of life at home. In our architecture, each sensor is able to interact and connect with Twitter. The system detects physical parameter changes in household appliances. These household appliances could be of any model and any manufacturer. A smart system joins data from sensors in the household appliances in order to take decisions. It connects each household appliance to the Internet, and thanks to the integrated information, we obtain a system with smart capabilities. We go beyond other centralized architectures that only forward information of sensors to Internet. Information of the sensors in our architecture is fused in order to notify the user with high level semantic messages when necessary.

The paper is structured as follows: Section 2 discusses the related literature. System architecture and its elements are described in Section 3. Section 4 explains the system operation, the protocol, smart decision algorithm and system notifications. The system performance is shown in Section 5. Section 6 draws the conclusions and describes future work.

## 2. Related Work

The idea of using existing electronics in smart home appliances and connecting them to the Internet is feasible nowadays thanks to the fast development of information and Internet technologies. Some electronics giants are selling various kinds of smart home appliances [9] and lots of electrical appliances are starting to be digitized and combined with network technologies.

These products are usually introduced as proprietary systems, which are only provided with the electrical appliances belonging to a specific brand. As an example, LG Electronics [10] has a full range of smart appliances, enabled by LG THINQ™ Technology, comprising five key features—Smart Grid, Smart Diagnosis™, Smart Access, Smart Adapt and Food Management. The system we present is able to be used on any electrical appliance without necessity of belonging to a specific brand.

Our proposal will also use social networks to deliver the appliances' knowledge. When connecting home appliances to the Internet, there are serious challenges that must to be addressed, among others, the need for security [11] and the need for interoperability among various technologies [12]. In [13] and [14], the authors presented intelligent refrigerator systems. In [13] a volume sensor is used. An extension of this work is to inform the status of the refrigerator through Short Message Service (SMS) using the Global System for Mobile Communications (GSM) network infrastructure. In [14] the system also keeps the track of the amount of empty space, having a LCD display screen that shows a message when the items are over. This intelligent refrigerator will find out the stock online via iInternet using IEEE 802.3U Ethernet technology.

However, in our implementation, many people can read the physical values obtained by the sensors via the Web. It does not matter the type of communication device used to access the published information.

Other proposals are based on the establishment of web services [1,15–17]. In [15], the authors propose an infrastructure based on web services using heterogeneous sensors and clients that interact using WS-Notification. The home network is dynamic and the hierarchy of events that can be published and subscribed have a dynamic structure. As a case study, they present an implementation for monitoring the health of an elderly adult. In [1], the same goal is followed. They propose a concrete architecture for a home in which the falls of the elder adult in a domestic environment are monitored. The architecture is implemented on a heterogeneous set of devices. The resulting information system is scalable as devices can join and leave the architecture dynamically. Also, the hierarchy of published and subscribed events is dynamic and may be modified at runtime, which allows them to evaluate it and draw conclusions on the feasibility of using service-oriented approaches in ubiquitous computing. In [16], a web service based approach to enable an evolutionary sensornet system is presented. The functionality and data provided by the new nodes are exposed in a structured manner, so that multiple applications may access them. The result is an inter-operable system where applications can share a common evolving sensor substrate. They prototyped an example application for home energy management, demonstrating how structured data and programmatic specification of functionality can be enabled in resource-constrained sensor nodes. The authors in [17] presented an approach to interconnect different sensors and actuator nodes in building control and monitoring systems. They present a web services-based approach to integrate resources constrained sensors and actuator nodes into IP-based networks. Finally, they showed by measurements that the system offers an acceptable performance given the limited computing power and memory constraints of the hardware platform.

In [18–21], web based smart homes are studied. In [18] a home automation via the Web based on eXtensible Messaging and Presence Protocol (XMPP) and Open Service Gateway Initiative (OSGi) technology is proposed. In particular, the authors propose a fault detection mechanism for digital refrigerators with the aim that the technical staff can do a fast diagnosis and respond remotely. The authors in [19] investigate the possibility of deploying a Web-based, energy-aware smart home, adapted to demand response. They carried out an initial technical study that denotes the feasibility of their approach. In the future, they plan to perform a small-scale deployment of their Web-based, energy-aware home infrastructure in real houses. A technical report to describe the architecture of a Web-based smart home framework is done in [20]. A fundamental element of the framework's architecture is the use of request queues. A flexible application-level solution for home automation is proposed, based on combining existing Web technologies and reliable Web techniques. They present a novel design of the framework, employing request queues for handling the interaction of tenants with their home devices, which enhances the overall performance of the system, offering reliability and fault-tolerance. Some authors propose reusing the principles of modern Web architecture to fully integrate physical objects to the Web and build an interoperable Smart Home. In [21] an application framework that offers support for multiple home residents concurrently is presented. They developed an application framework, based on REST architectural style that offers a uniform, efficient, and standardized way of interacting with information appliances. They show how scalable flexible Web applications can be built using the Web as application layer. This Web application can serve a large number of simultaneous residents who are able to fully automate their houses.

Our proposal conveys to the user of the appliance (neither the technical staff nor the manufacturer) that the refrigerator is “alive” (not only that it has a fault). As social networking on the Web has become an integral part of our lives, our proposal also communicates directly with the user via a social network that is more extensible that one that communicates only with the technical staff and manufacturer.

The authors in [22–24] propose utilizing existing social networking infrastructures such as Facebook and their Web-based APIs in order to integrate Smart Homes and Farms to the Web, offering social status to physical devices.

On the one hand, we do not only publish isolated sensed values but the combination of several sensed values that provide more information about the household appliance. A previous processing of the sensed values makes more understandable about what is really occurring at that moment and reduces false alarms. On the other hand, Facebook is more focused on sharing information with friends, while Twitter is quicker to post news and easier to be viewed by the users, which is the main purpose of our system. We were looking for an instant messaging social network that allows posting of different topics (which in our case can be different electric household appliances) and allows monitoring any issue very fast. Our proposal publishes on Twitter Network since we think that microblogging services are appropriate to do our environment available online. Our system tweets to those users that are interested in reading the sensed values. Unlike Facebook, the social networking platform selected by the authors in [22–24], Twitter does not have a stable open Application Programming Interface (API) that provides rich possibilities to application developers. Twitter has relatively few features when compared to Facebook.

Other proposal based on publishing on social networks is the one shown by Rahman *et al.* [25]. They show how sensor devices can monitor different daily activities of a human body by forming a body sensor network. The captured sensory data were delivered to one's community (COI) which can also be regarded as one's social network. In this paper, they propose a framework that creates an overlay network to create a secure communication passage for dynamic sensory data communication among users belonging to the same COI. We have also found The WiFi Body Scale [26], which monitors changes in the weight of the user and sends him/her graphs to an iPhone mobile application. It has included others features like sharing the progress on a blog, Facebook or Twitter.

A model that uses Twitter as a communication backbone to collect information from geographically sparse sensors and publish sensory information to enhance the management of sensor networks is presented in [27]. The presented work is in its early stage.

The design and implementation of a crowd-sourced sensing and collaboration system over Twitter using smartphones is presented in [28]. The system is applied in two contexts: a crowd-sourced weather radar, and a participatory noise-mapping application. The experimental results show that Twitter can provide a publish-subscribe infrastructure for sensors.

After having reviewed the state of the art in this topic, we can see that there is no low-cost system, such as the one proposed in this paper, which can be used in any type of electrical household appliances and it is able to gather data from them and posts the information in Twitter. Moreover, none of the related works have included a smart system that makes decisions based on the gathered sensed data, which makes our system an innovative product.

## 3. System Architecture

In this section we explain the architecture of the system and detail the operation of its elements.

### 3.1. System Overview

The main purpose of our system is to gather information from the electrical household appliances and post what is happening on the Twitter Social Network. In order to develop our system, we will assume a regular house with an Internet connection. It could be a router allowing Internet access to all devices in the Local Area Network (LAN) or a Personal Computer using a modem.

**Figure 1.**
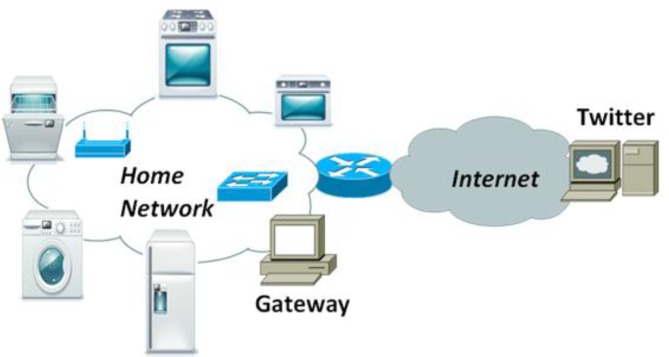
Elements of the system.

We will assume that there will be one computer that has Internet access and it is reachable by any device in the LAN (wired or wireless). From now on, we are going to call it Gateway. This gateway will be able to know what is happening in each electrical household appliance, based on the received information, and it will be able to post some information, taking into account the data from the appliances, in Twitter Social network. There will be several physical sensors that are able to measure some information from the electrical household appliances. The type of sensors used in each case depends on the type of information needed to gather from each appliance. Moreover, each electrical household appliance will have a Local Sensor Host (LSH), which is a device developed by us and will be later detailed, that will be able to take information from the appliances and forward it to the gateway though the wired or wireless LAN. The elements of the system are shown in Figure 1. It consists of a series of electrical household appliances that have connected a LSH which at the same time are connected to the LAN wired or wirelessly.

**Figure 2.**
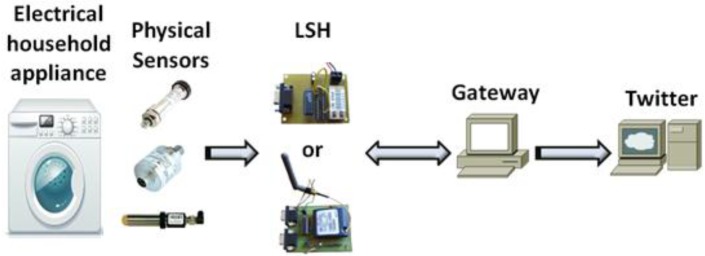
System architecture communication.

The operation of the system is as follows. There are some sensors placed inside the electrical household appliances that are gathering some physical information such as temperature, humidity, pressure… These sensors are connected to the LSH using a serial connection (RS-232) or transistor-transistor logic (TTL) technology. LSHs are connected to the LAN and allow connections from a computer in the same LAN. The gateway takes a decision according to the received data and posts the appropriate advice to Twitter. Figure 2 shows the system architecture communication. It also shows the communication direction.

### 3.2. Local Sensor Host

The aim of the LSH is to let any device of the network collect the sensed data from the electrical household appliance. In order to develop the most appropriate device, we considered that the device must be able to take information from several sensors. For that reason we included recommended standard serial connections in our device: RS-232 and TTL. Moreover, we also took into account that we might access the LAN wired or wirelessly, so we developed two types of LSH, one with IEEE 802.3u for the wired connection and another one with IEEE 802.11g for the wireless connection.

#### 3.2.1. Wired LSH

For the wired connection we used XPort device, from Lantronix [29]. It has a CPU based on the DSTni-EX enhanced 16-bit ×86 architecture (48 MHz or 88 MHz, depending on the model). It has a memory of 256 KB SRAM, 512 KB flash and 16 KB boot ROM.

On one side it has a serial interface which supports data rates from 300 bps to 921,600 bps. The serial interface allows characters of 7 or 8 data bits, the parity can be odd, even or none, the stop bits can be 1 or 2, it supports control signals DTR/DCD, CTS, RTS and flow control XON/XOFF (by software), RTS/CTS (by hardware), and none. The network interface has RJ45 connectors and supports Ethernet 10Base-T and 100Base-TX (Auto-Sensing). It also has link, activity and full/half duplex indicator, and indicators that let us know the type of connection (Ethernet or Fast Ethernet).

Xport device supports the following protocols: TCP/IP, User Datagram Protocol (UDP)/IP, Address Resolution Protocol (ARP), Internet Control Message Protocol (ICMP), Simple Network Management Protocol (SNMP), Trivial File Transfer Protocol (TFTP), Telnet, Dynamic Host Configuration Protocol (DHCP), Boot Strap Protocol (BOOTP), Hypertext Transfer Protocol (HTTP), and AutoIP. Moreover, it has an internal web server with a storage capacity of 384 KB for static web pages and Java applets. Web pages are compressed. The web server supports GET and HEAD methods, but it does not support the POST method, or any “server side” required processing. It supports 256-bit Advance Encryption Standard (AES) encryption for secure communications. Moreover it has three programmable Input/Output pins.

Newer versions such as XPort AR are able to deliver true IEEE 802.3af compliant pass- through Power over Ethernet (PoE) to edge devices using both Ethernet pairs. They have 1.25 MB internal directory-/file-based file system, they have a Common Gateway Interface (CGI)/Cascade Style Sheet (CSS) based web server and support Secure Socket Layer (SSL).

**Figure 3.**
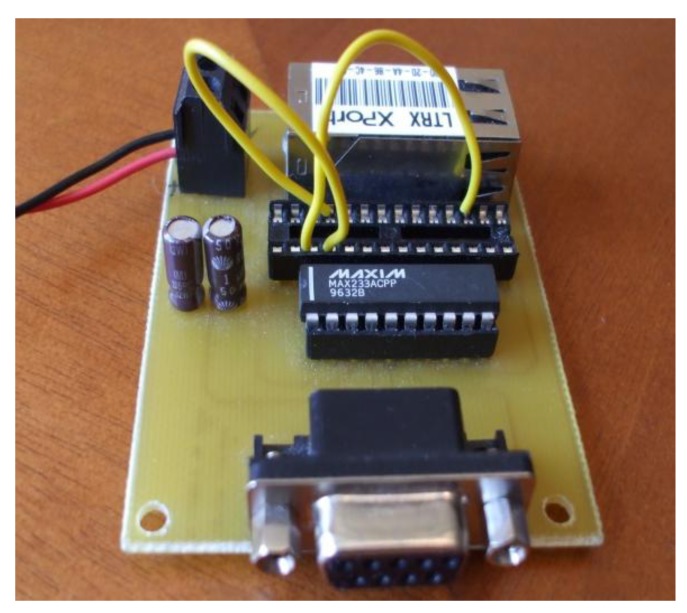
Wired LSH.

Our device is able to receive requests from the network interface and let us gather through the IP network all information received from the RS-232. It allows remote command and control of the sensors placed in the electrical household appliances. Our deployment is shown in Figure 3.

#### 3.2.2. Wireless LSH

For the wireless connection we used a MatchPort b/g device, from Lantronix [30]. It has a Central Processing Unit (CPU) Based on the DSTni-EX ×86 architecture. It has an on-chip memory of 256 KB zero wait Static Random Access Memory (SRAM), 2,048 KB flash and 16 KB boot Read Only Memory (ROM).

On one side it has two serial interfaces which support data rates from 300 bps to 921,600 bps. Both serial interfaces allow characters of 7 or 8 data bits, the parity can be odd, even or none, the stop bits can be 1 or 2, and support control signals DTR/DCD, CTS, RTS and flow control XON/XOFF (by software), RTS/CTS (by hardware), and none. The wireless network Interface meets IEEE 802.11 b/g standard in the frequency range from 2.412 GHz to 2.484 GHz. It allows automatic fallback data rates from 54 MBps to 1 Mpbs. Without the antenna gain it has an output power of 14 dBm +2.0 dBm/−1.5 dBm with a Maximum Receive Sensitivity −91 dBm @ 1 Mbps. The modulation techniques that can be used by the device are Orthogonal Frequency Division Multiplexing (OFDM), Direct Sequence Spread Spectrum (DSSS), Complementary Code Keying (CCK), Differentially encoded Quadriphase Shift Keying (DQPSK), Differential Binary Phase Shift Keying (DBPSK), 64 Quadrature Amplitude Modulation (QAM), and 16 QAM. It has an average consumption of 740 mW when it is active and 250 mW when it is inactive. Its peak supply current is 360 mA when transfers data. Moreover, it has implemented IEEE 802.11i-PSK with AES-CCMP Encryption, WiFi Protect Access (WPA)—Phase Shift Keying (PSK), Temporal Key Integrity Protocol (TKIP) Encryption, 64/128-bit WEP, and optional 256-bit AES end-to-end encryption.

**Figure 4.**
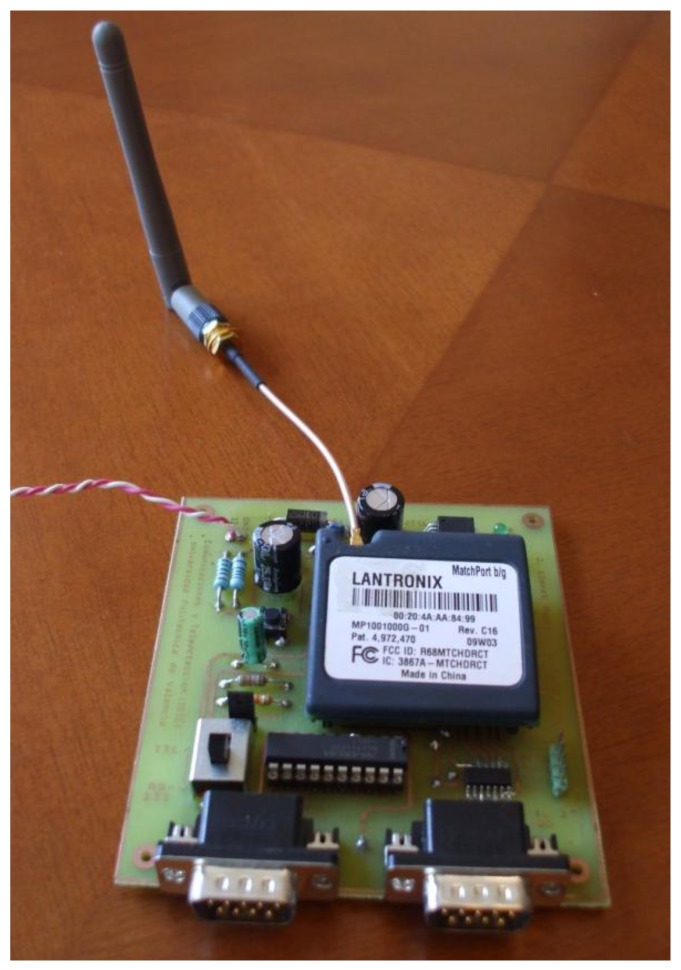
Wireless LSH.

Matchport device supports the following protocols: TCP/IP, UDP/IP, ARP, ICMP, SNMP, TFTP, Telnet, DHCP, BOOTP, HTTP, and AutoIP. Moreover, it has an internal web server with a storage capacity of 1.2 MB. It has 8 General Purpose Input/Outputs (GPIO). Our deployment is shown in Figure 4.

### 3.3. Sensors

Initially we could use any sensor available in the market with TTL voltage level or RS232 connection to connect with our LSH. Table 1 shows some of the available sensors. TTL lets us connect a sensor to any microcontroller. If we wish to connect a sensor to a computer using a RS232 port, we will need to use a level converter circuit such as the MAX232 or similar. In order to avoid the use of integrated circuits to do the connection, we can use discrete components to make a scheme that allows the use of RS-232 connections without the MAX232 circuit [31]. Once the connection is established, the connection parameters are fixed. They let the communication starts using the sending and receiving data functions in the serial port.

The following list shows some examples of measurements that can be obtained with some of these sensors:

- Light. For example, we can check if the fridge is open. We can use it instead of the fridge opening sensor if it is broken. We can also detect the failure of the electric power.
- Pressure. For example, it can be used to check if the trays and bottles are full or empty.
- Presence (acoustic sensor). For example, it may be used together with the pressure sensor to check if the fridge is full or empty.
- Heat. It can help to increase fridge temperature. We can also check if the food is well preserved depending on the amount of food in the fridge.
- Humidity-Temperature. It can be used for humidity optimization depending on the type of food that is currently in the refrigerator.
- Acidity Sensors are electro-chemical sensors that react to pH changes. They can be used to monitor the food quality.

In our system we use the sensor which already is included on the fridge. Our device is connected to the temperature and the open door sensors. It lets us increase the performance of our system because we minimize the number of connected sensors. We have also selected two additional sensors: acoustic and pressure. Pressure sensors also incorporate temperature sensors that let us measure temperature on each tray. Temperature sensors are useful to improve the quality of the stored food. The temperature is not the same in all trays, so with temperature sensors placed in different trays we will optimize the fridge performance.

The established configuration lets us know the available food without the necessity of monitoring how much food comes in and goes out of the fridge. We have distributed the sonar sensor as follows: there are four pressure sonar sensor under the main trays and three additional sonar sensor under the door's trays. There are four acoustic sonar sensors upper each main tray and three additional acoustic sonar's upper each door's tray.

Main trays' sensors let us monitor the availability of food. Acoustic sensor on each tray measures how far is the food on this tray. The pressure sensor lets us measure the weight of each tray. A combination of both determines the quantity of food we have in our fridge. A fundamental fact of our research is the combination of data from various sensors to obtain the correct information. Having only the pressure sensor is not enough. Depending on the type of food, there could be more or less weight (more or less pressure) on each tray. For example, vegetables do not have the same weight as milk or meat. We must complete these data with the quantity of food on each tray. Acoustics sensors let us know if there are elements on each tray. Thanks to both sensors we know which products are missing in our fridge.

We have noticed that if we use some fridge organization, for example: on tray 1 the dairy products, on tray 2 the vegetables, on tray 3 the protein products, and so on, the system performance is improved considerably.

Door sensors let also check the fluid pressure on a membrane controlling the availability of fluid. It can provide the availability of drinks.

After analyzing sensors characteristics we have selected the SRF02 Ultrasonic range finder sensor and the 33X High Accuracy Digital Output Pressure Sensor. Each one has been connected using a RS232 port. Next we present some details about the selected sensors and the way we have worked with them.

- SRF02 Ultrasonic range finder. The SRF02 is a single transducer ultrasonic rangefinder in a small footprint PCB. It features both I2C and serial interfaces. The serial interface is a standard TTL level UART format at 9,600 baud and it may be connected directly to the serial ports on any microcontroller. Up to 16 SRF02s may be connected together on a single bus, either I2C or Serial. The SRF02 includes the ability to send an ultrasonic burst on its own without a reception cycle, and the ability to perform a reception cycle without the preceding burst. SRF02 uses a single transducer for both transmission and reception so the minimum range is higher compared to other solutions. The minimum measurement range is around 15 cm. The power supply is 5 V and current average consumption is 4 mA. The dimensions are: 24 × 20 × 17 mm high and the weight is 4.6 g. We have connected seven sonars, one in each tray and three in the side compartments (door trays). To communicate with the SRF02, it is necessary to send two bytes with the address of the SRF02 (factory default is 0) and the command. The default shipped address can be changed by the user to any of 16 addresses 0, 1, 2, 3, 4, 5, 6, 7, 8, 9, 10, 11, 12, 13, 14, or 15. Therefore, we can use the seven sonars without problems. We have changed the sonar's addresses to access to the different measurements. To measure a new data a command has to be typed on the command register and then, we wait a while (about 65 mS) to read the registers 2 and 3.
- 33X High Accuracy Digital Output Pressure Sensor. The 33X digital pressure sensor incorporates silicon strain gauge sensing technology and digital characterization techniques to produce a highly accurate pressure sensor over a wide temperature range. The live pressure reading can be measured or a data logging procedure can be set up for recording pressure data at set intervals. It is based on the stable, floating piezo resisitive transducer and the newly developed XEMICS micro-processor with integrated 16 bit A/D converter. Temperature dependencies and non-linearities of the sensors are mathematically compensated. The pressure ranges are from 1 up to 1,000 bar (15–15,000 psi) gauge, absolute or sealed gauge. The output signal can be obtained from USB, RS232 or RS485 digital interface for pressure, temperature and programming. This sensor lets connect up to 16 digital pressure sensors on one serial bus connection, measure/log pressure and temperature simultaneously. We use seven pressure sensors. The 33X digital pressure sensor also incorporates a temperature sensor which is located very closed to the diaphragm in contact with the media. . This temperature sensor is used to monitor if the fridge is open too much time, if the temperature is higher or lower than needed to maintain the food. It has high precision pressure transducer with an accuracy of 0.05% full scale including temperature errors over +10 to +40 °C.

**Figure 5.**
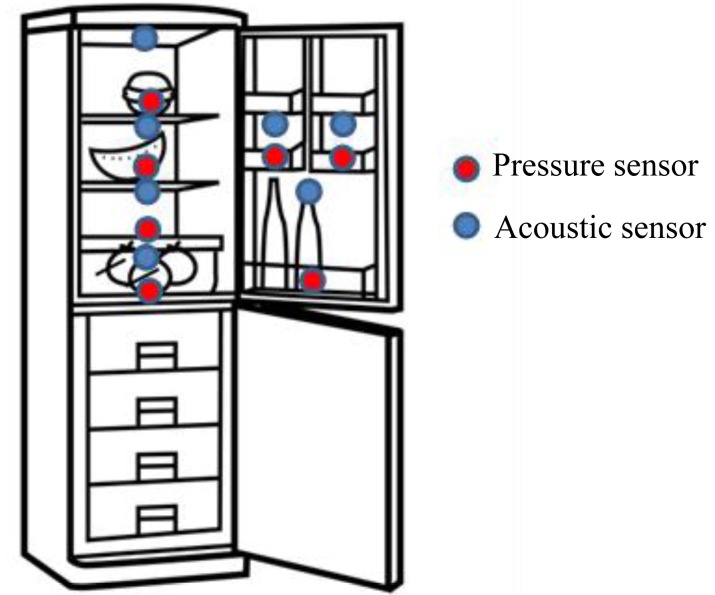
An example of a refrigerator with sensors.

Figure 5 shows a refrigerator with the proposed sensors. Pressure sensors also include temperature sensors. All sensors are connected with the Wireless LSH with wire.

### 3.4. Gateway

The Gateway runs in a Personal Computer which allocates:

- A Web Server that is in charge of storing the sensor values transmitted by the LSH. One of the objectives of this Web server is to store historic values of sensor measurements (the amount of memory of the XPort AR device is very limited to support them). The reason to store historic values is that we can implement robust fusion software using them.
- Communication software which receives the values of the sensors sent by the LSH and sends them to the Web Server allocated in the Personal Computer. This software is called Application Level Gateway (ALG). ALG is in charge of sending the notifications to Twitter, so it translates the sensor values received into a set of words that can be understood by the humans. It performs the following basic actions: (a) A HTTP GET request to the LSH in order to gather the values of the sensors, (b) the translation of sensor values in a set of words, and (c) the storing of the set of words in a file inside the Personal computer where is running the ALG.
- User registration software. This software is in charge of registering the users that want to be notified when alarm events occur in our electrical household appliances. The main information to be registered is the mobile telephone of the user and its Twitter account. This software is also in charge of managing registered users: delete or modify their data (this can be done by an administrator or the system can let the users modify their own data filling some data in a simple web page).
- Alarm software and notification of special events. This software is in charge of reading the set of words and decides when to tweet an alarm to Twitter.

Twitter publication software. This software is in charge of receiving an alarm (alarm software) and to publish the associated information in Twitter. To do this we must use the standard HTTP connection protocol to Twitter (Twitter connector).

### 3.5. Analytical Model

**Figure 6.**
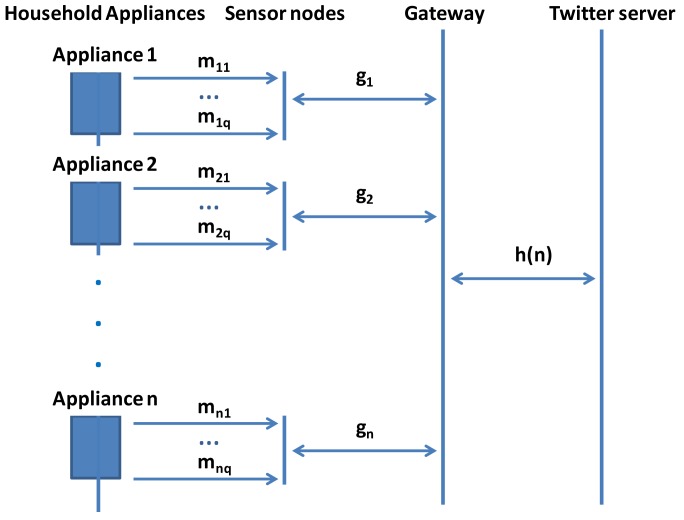
Analytical view of our system.

Let a set V of house appliances be in the network, being n = |V|, with j physical sensors (where j = 1, 2, …, q) for each i household appliance (where i = 1, 2, …, n). In order to make our system simpler, we will assume that each household appliance is formed by only one sensor node connected to all physical sensors inside the household appliance. Let us suppose that the sensor nodes placed in every appliance are able to gather an average value of m_i_ messages per second from the physical sensors for each i appliance. After gathering this information, sensors will only exchange messages with the gateway when they gather values that are different from the previous ones. Let us suppose that sensor nodes exchange with the gateway an average value g_i_ messages per second for each i sensor node. Finally, the gateway will only exchange messages with the Twitter server when a decision is taken. Let us suppose that the gateway exchanges an average value of h(n) messages per second with the Twitter server (the average value depends on the number of household appliances at home). Figure 6 summarizes the explained procedure.

The number of messages (*M*) delivered in our system is given by expression (1):

(1) $$M=\sum_{i=1}^{n}\sum_{j=1}^{q}m_{i,j}+\sum_{i=1}^{n}g_{i}+h(n)$$

where *n* is the number of household appliances (there is one sensor node per household appliance) and all other parameters are defined previously.

**Figure 7.**
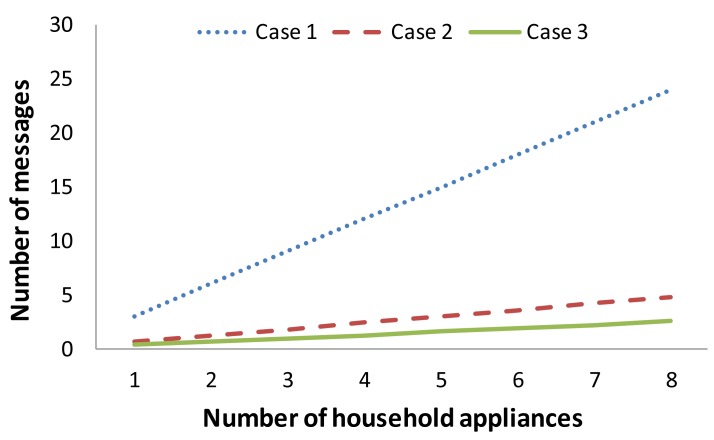
Number of messages in our system as a function of the number of household appliances.

Now, we are going to see how this equation behaves for a regular home as a function of the number of household appliances (there could be many appliances, e.g., refrigerator, dishwasher, washing machine, dryer, microwave, oven, *etc.*). Each one has one sensor node with three physical sensors (it is a good estimation since every household appliance can be controlled well sensing only three parameters). Figure 7 shows the number of messages exchanged by the whole system as a function of the three cases: (1) when all sensor nodes gather one message per second from each physical sensor (it can be defined by hardware), but all physical parameters change their values with an average time of 7,200 seconds (2 hours) so a message is sent from the sensor node to the gateway, and only one notification is sent every 18,000 seconds (5 hours) from the gateway to the Twitter server, (2) when all sensors gather 1 message every 5 seconds from each physical sensor, but all physical parameters change their values with an average time of 3,600 seconds, and only one notification is sent to the Twitter server every 10,800 seconds (3 hours), (3) when all sensors gather 1 message every 10 seconds, but all physical parameters change their values with an average time of 60 seconds, and only one notification is sent to the Twitter server every 18,000 seconds.

We can see in Figure 7 that the system has a larger number of messages when the time to gather data from the physical sensors is shorter. It will not affect to the IEEE 802.3u or IEEE 802.11g network, but it will affect to the power consumption of the whole system. Although physical sensors change their values more frequently in case 3 than in case 2 because in case 2 sensed data are gathered twice every 10 seconds, the number of messages sent to the system is higher.

**Figure 8.**
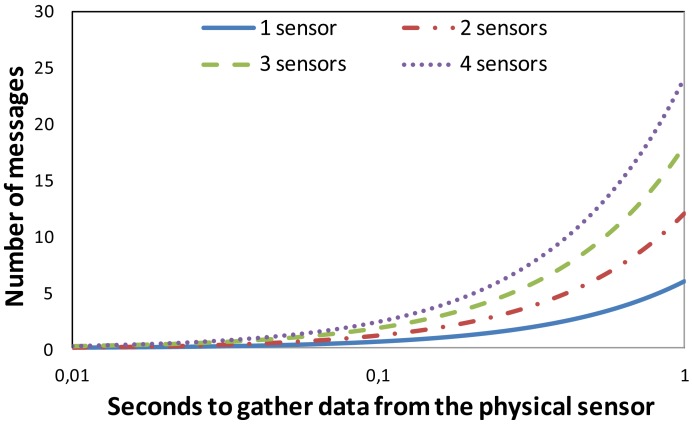
Number of messages in our system as a function of the number of household appliances.

In order to study the system behavior when we vary the number of seconds to gather a sensed value from the physical sensor, we provide Figure 8. In this case there are six household appliances and we can see the number of messages sent to the network when these appliances have 1, 2, 3 and 4 physical sensors and we vary the time to gather the data from them. We can see that there is an exponential increment in the number of messages when the number of sensors increases.

## 4. System Operation

In order to gather the measurements taken from the sensors, we can use two procedures. The first one is the active procedure, where the physical sensor pushes the sensed values to the gateway continuously just after they have been taken. The second one is the proactive procedure, where the physical sensor pushes the sensed values when they are pulled by the gateway. We can develop both types of data gathering procedures, but in order to avoid overloading the network when there are many electrical household appliances connected, we decided to use the proactive procedure, where the gateway requests the LSHs of the network. During the system performance evaluation we will see the difference between having a gateway requesting the data *versus* having LSHs continuously sending data to the network.

### 4.1. Protocol between the Gateway and the LSH

**Figure 9.**
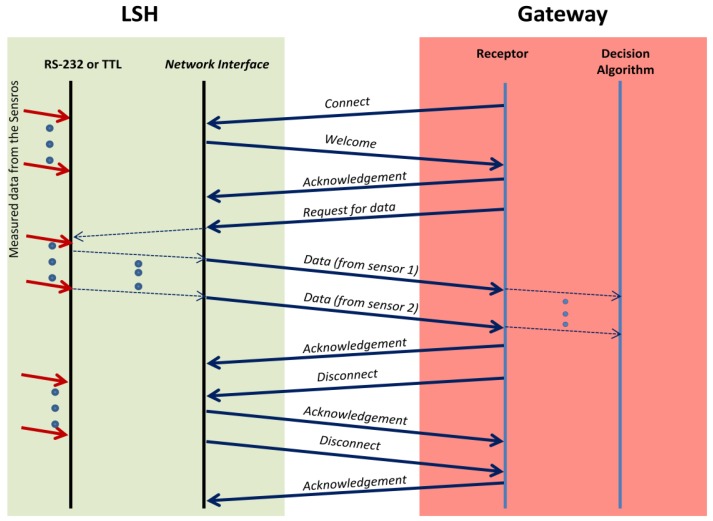
Communication protocol between LSH and the gateway.

We have developed a three-way transmission protocol in order to gather the data from the sensors placed in the electric household appliances. The gateway connects each LSH using a connection oriented protocol. First it sends a *Connect message* to the LSH. The LSH replies with a *Welcome message* in order to indicate that it is active. Then, the gateway sends an *Acknowledgement message*. After this, the gateway requests the data to the sensors using the *Request for data message*. LSH sends the values obtained directly from the sensors through the RS-232 connection, by using *Data messages*. Once the Gateway receives last *Data message* (there should be very few messages because there are not too much sensors and sensed data should have very few Kbytes), it sends an *Acknowledgement message*, and, after it, a *Disconnect message*. LSH will also send an *Acknowledgement message* and a *Disconnect message*, which ends the connection. Finally the gateway acknowledges last disconnection message. We can see the explained protocol in Figure 9.

### 4.2. Gateway Decisions

**Figure 10.**
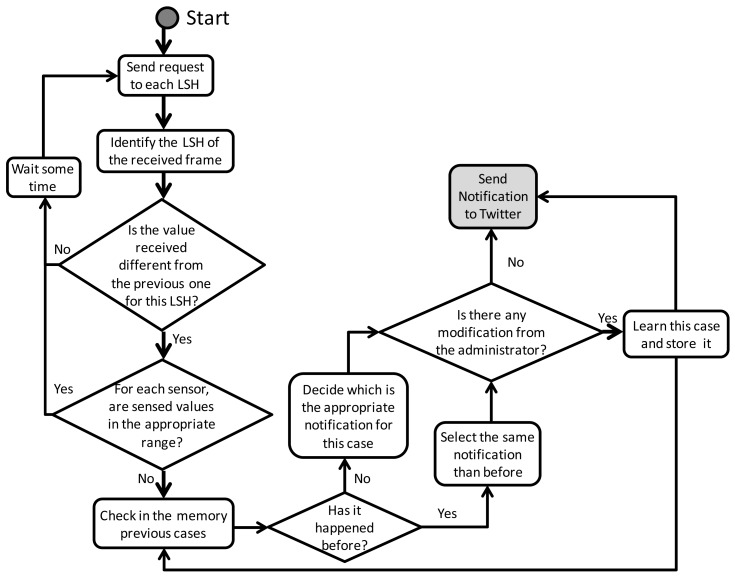
Gateway decision algorithm.

The gateway takes its decisions and sends the notifications to Twitter according to the data received from the sensors. In order to perform this task we have designed a smart decision algorithm which also learns from previous experiences and from the decisions taken from the administrator of the system. The algorithm is shown in Figure 10. Initially the gateway sends a request to each LSH. When it receives the frames from the LSH, it identifies which electrical household appliance the LSH is connected to. Then, it looks inside the values received and compares them with the previous values for the same LSH. If there is no difference, it waits for the next request round, but if there is something different, its first action is to see if the sensed values are inside their appropriate range. If they are inside their range, no action is taken and it waits for the next request round, but if they are outside of their range, some action is taken. First it checks in the memory the previous cases in order to see if it has happened before. If it has happened before, it decides to send the same notification, but if it has not happened before, it decides the appropriate notification for this case based on a predefined set of rules. Whether it has happened before or not, there could be a decision taken from the system administrator which will be learned from the system and saved it to be taken into account for future cases. After this, the gateway posts the notification to Twitter.

### 4.3. Notifications

In this section we present some notifications of different appliances. We have reviewed some typical electronics data sheets to elaborate the list below. As an example, for each appliance, we present the different sensors that can be used and the notifications that can be issued to the LSH. Let us take into account that a smart house (considering this list) can notify the user instantly the state of several appliances.

In Tables 2–6 we describe examples of notifications that the appliances could alarm. The following example of processing for the Refrigerator can be extended to the rest of appliances. That is to say, the Xport receives sensory information of SRF02 door opening, light, Ultrasonic range finder and 33X High Accuracy Digital Output Pressure sensors. Using recommendations given in Section 3.3 about the fridge organization we present the corresponding notifications in Table 2. For the rest of appliances we proceeded in the same way.

The vibration sensor is the most used for the washer appliance. Apart from humidity and vibration sensors, the temperature sensor can always be used for the dryer appliance. The clothes have been drying in 1 hour! can be notified in the same way we indicated for the washing appliance. The temperature sensor is the most used one for this appliance.

### 4.4. Protocol to Post Notifications in Twitter

**Figure 11.**
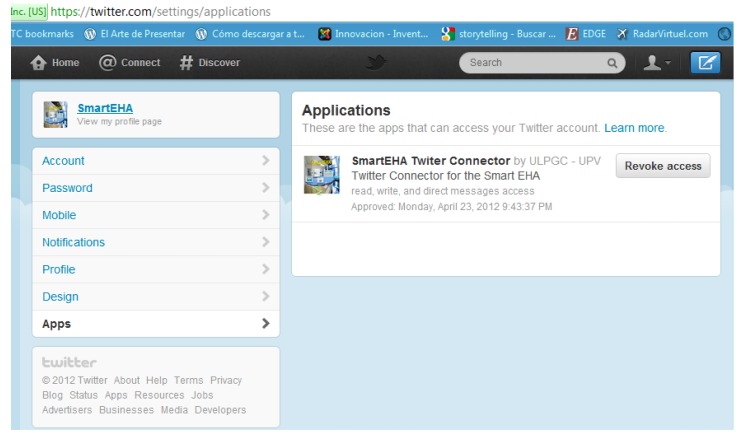
Twitter connector application for SmartEHA.

**Figure 12.**
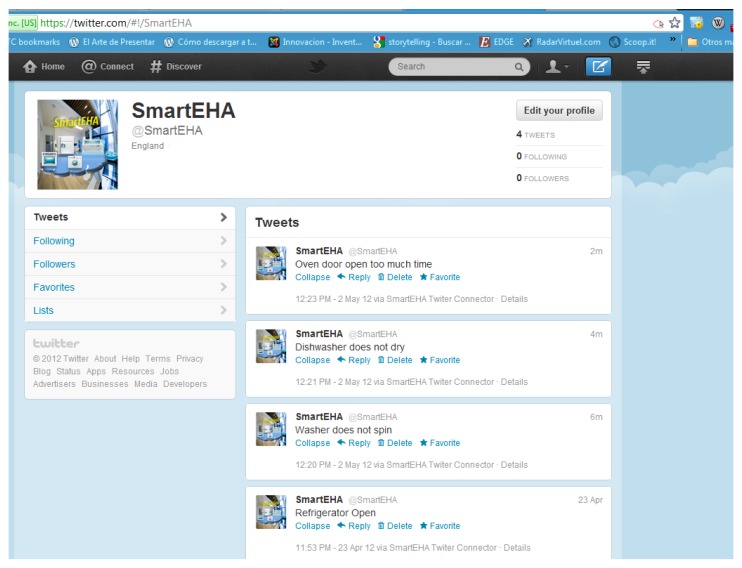
Several tweets in the SmartEHA account in Twitter.

To publish the above alarms in Twitter it is necessary to set up a new user account for the electrical household appliances. The process to register them in Twitter is as simple as to register an account with a name. We have signed up our system in Twitter with the user account @SmartEHA (Smart Electrical Household Appliances). SmartEHA's tweets enable the followers (mainly their users) find out what's happening instantly with them receiving notifications directly in their mobiles or in the desktop computer. Although tweets are publicly visible by default we have restricted message delivery to its followers (a family living in that house). Figures 11 and 12 show some captions taken from the SmartEHA account. Figure 11 shows the Twitter connector application for the SmartEHA account in Twitter. The name of the application is shown on the right hand of the figure. In Figure 12 several tweets show the generated alarms. These alarms are different depending on which electrical household appliance generates them.

The first step we must do is to register the account for the SmartEHA in Twitter. Once this has been done then we must develop a Twitter Connector for this account. This consists on implementing a Twitter application that runs in the Twitter web site. In this process, we will obtain the following four connecting values that are crucial to manage secure communications: consumer_key, consumer_secret, user_token and user_secret. The two first ones are used for the identification of the Twitter Connector (Twitter application), and the two last ones are used for the identification of the account of the SmartEHA. Any Web application that wants to publish information in our SmartEHA account must know these values.

Let us remember that the Web components software in the Home PC that monitors the reception of sensors' values from the LSH makes the fusion of data, controls the alarm warnings and publishes the alarm tweets in Twitter.

There are three main components inside the Web server running in the Home PC: Receptor, Alarm Controller and Twitter Publisher. The Receptor is in charge of periodically receiving the sensors' values from the LSH. These values are stored in a file: In this way we can decouple the period of sense in the sensors and the speed of fusion made in the Alarm Controller component. The actions of the Alarm controller are basically two: The fusion of sensor data and the generation of alarms. When an alarm is generated, the Publisher will receive a notification. Then the Twitter Publisher component will issue the publication in Twitter. For each publication, the Twitter Publisher component will issue a secure authentication using OAuth [48] with the Twitter Connector. OAUth is used from the Representational State Transfer (REST) Application Programming Interface (API) [49] that is based on HipertText Transfer Protocol Messages (HTTP). This API uses a Hash-based Message Authentication Code (HMAC) Secure Hash Algorithm named SHA-1 which produces a 160 bit message digest. This authentication message uses the consumer_key, consumer_secret, user_token and user_secret values to cipher the communications. The importance of this is that communications and publications will be done in a secure way. Once it receives the appropriate validation (cookie) it will then confirm the secure registration and it proceeds to send the alarm tweet that will be received by the SmartEHA Twitter Account. This publication fundamentally is based on using a HTTP POST message named statuses/update in the REST API.

**Figure 13.**
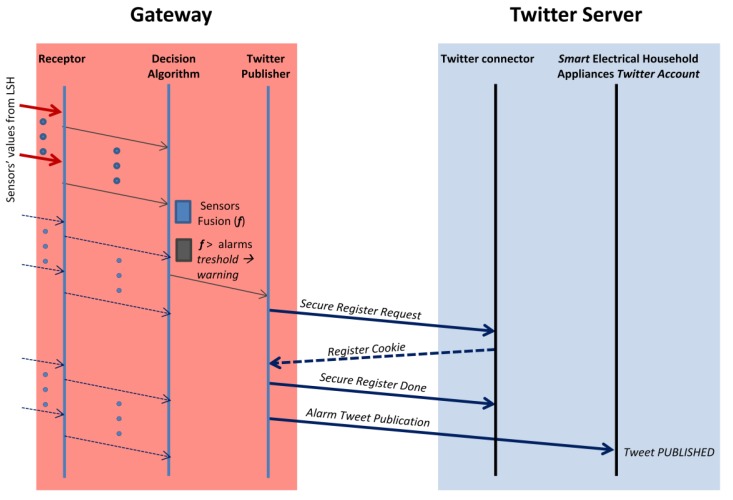
Communication protocol between Web Server and Twitter.

In Figure 13 we present the main ideas of the overall protocol. While the Receptor is receiving the sensors' values, the Alarm Controller is processing their fusion in order to generate the appropriated alarms. If an alarm is generated then the Twitter Publisher will first issue a secure authentication in the Twitter connector (basically a one way handshaking process consisting in three messages) and then it will send a POST HTTP message in which the body of the text of the concrete alarm will be published in the SmartEHA account.

Let us note that with this process we are taking into account the worst case for the delay of publication (we could first authenticate the Twitter Publisher and then POST a set of tweets). But this method assures that no time connection will be produced in case the interval between two consecutives tweets is very high.

## 5. System Performance

In order to test the system performance we have split the system into two parts. The first part tests the system performance from the LSH devices to the gateway, and the second one tests the system performance from the gateway to the Twitter server.

The performance test will be different for each part. This is because each part has different network performance requirements. In the LAN part we will take into account the encapsulation of sensors data in HTTP/TCP/IP. It could be probable that TCP incurs in a big overhead if it sends very small data values inside big packets. Moreover, the performance of the wired and wireless networks may be affected when there are many electric household appliances. Furthermore, the performance of WiFi disconnections may affect the quality of alarms. That is, we have to compare the wired and wireless local area networks in order to know if there is a big difference between them for this study case. The Wide Area Network part must also be tested because it does not depend on the customer and it cannot be controlled. In this case we will measure the latency of publishing data in the Twitter Social network.

### 5.1. Performance Test with the Electrical Household Appliance

The first measurement we have performed is the power consumption of the LSHs. Although we assume that usually LSHs are plugged in, we also thing that it could be useful to have the LSH powered by batteries or solar panels in some special cases. Table 7 shows the comparison table. We can see that Xport is powered with more voltage than Matchport, but Matchport consumes more current on the average.

The same sensors and very similar notifications can be used for a microwave appliance. In fact these two appliances can be used in only one machine in practice. Now we are going to show how our system performs in active mode and proactive mode. Our main purpose in this section is to show that on the one hand there is a low overload both in the LSH and in the gateway, so any type of Personal Computer can be used for this purpose, and, on the other hand there is a low bandwidth wasted, so we can add dozens of household appliances at home without being concerned about this limitation.

#### 5.1.1. Test of Data Gathering in Proactive Mode

**Figure 14.**
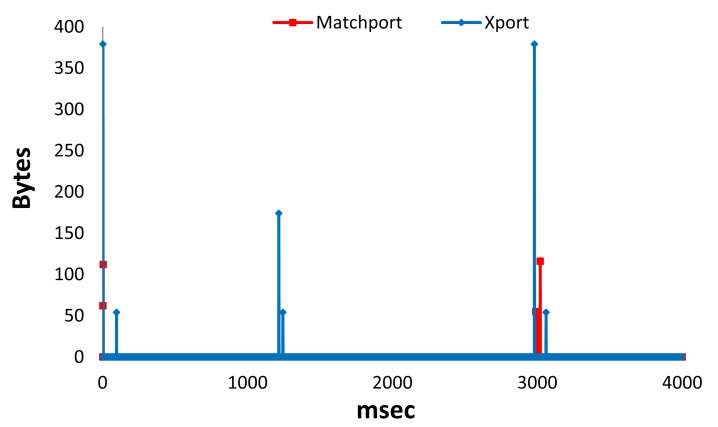
Bytes received per millisecond in proactive mode.

In this subsection we show the performance of the system when the gateway connects with a LSH that gathers the information from the physical sensors and disconnects for both types of LSHs (wired and wireless). Figure 14 shows the number of bytes received per millisecond for Matchport LSH and Xport LSH during 4 seconds (the process of gathering data only needed 3 seconds). We can see that there are higher peaks in Xport LSH. We have obtained an average number of bytes per millisecond of around 0.10 for Matchport LSH and 0.27 for Xport LSH (a number almost three times higher). Moreover, the highest peak for Xport LSH was 379 bytes, while the highest peak for Matchport LSH has been 116 bytes (almost 3.3 times higher).

**Figure 15.**
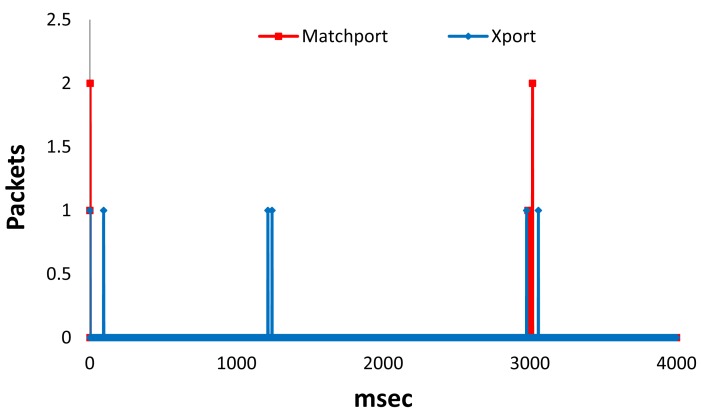
Packets received per millisecond in proactive mode.

In Figure 15 we can see the number of packets received per millisecond for Xport LSH and Matchport LSH in proactive mode during 4 seconds. We can see that there are higher peaks in Matchport LSH than in Xport LSH. We have obtained an average number of Packets per millisecond of around 0.0018 for Matchport LSH and 0.0015 for Xport LSH (almost the same value). Moreover, the highest peak for Xport LSH was 1 Packet, while the highest peak for Matchport LSH has been 2 Packets.

#### 5.1.2. Test of Data Gathering in Active Mode

**Figure 16.**
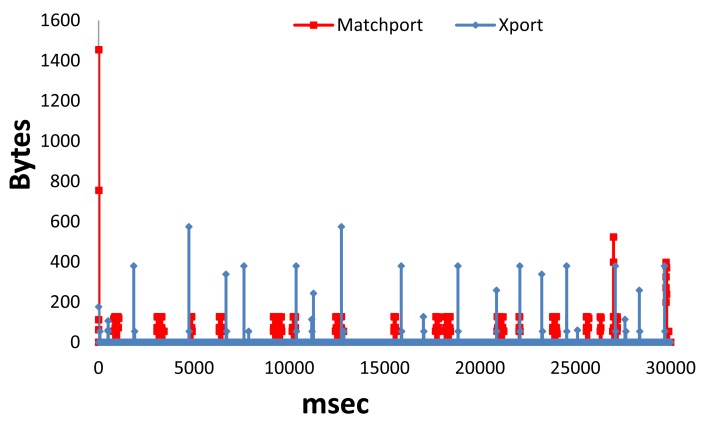
Bytes received per millisecond in active mode.

This subsection shows the performance of the system when the LSH is continuously sending the sensed data to the gateway for both the wired and wireless LSH. We have tested 30 seconds in each case in order to show the behavior of this mode and show its performance. The number of bytes received per millisecond for Matchport LSH and Xport LSH is shown in Figure 16. We can see that there are high peaks in Matchport LSH but there are more peaks in Xport LSH. We have obtained an average number of bytes per millisecond of around 1.05 for Matchport LSH and 0.26 for Xport LSH (around 4 times higher). Moreover, the highest peak for Matchport LSH was 1,454 bytes, while the highest peak for Xport LSH was 574 bytes (around 2.5 times lower).

**Figure 17.**
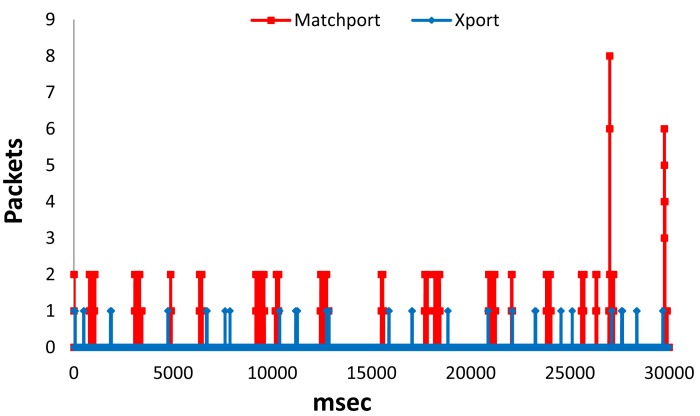
Packets received per millisecond in active mode.

Figure 17 shows the number of packets received per millisecond for Matchport LSH and Xport LSH in active mode during 30 seconds. We can see that there are higher peaks in Matchport LSH than in Xport LSH. We have obtained an average number of Packets per millisecond of around 0.0151 for Matchport LSH and 0.0015 for Xport LSH (10 times lower). Moreover, the highest peak for Xport LSH was 1 Packet, while the highest peak for Matchport LSH has been eight Packets.

We have observed that the average number of bytes per millisecond is between three and four times higher in Matchport LSH than in Xport LSH in both modes. Taking into account the number of packets per millisecond, we can see that there are higher peaks in Matchport LSH than in Xport LSH in both modes.

### 5.2. Performance Test to Post Notification in Twitter

We are interested in showing that the publication delay of SmartEHA's alarms in Twitter and their lecture notifications to the final user are very slow. This will allow the users to instantly be informed about these alarms.

To test the Twitter Connection we choose a portable computer with a Core Duo processor at 2.26 GHz with 4 GB of RAM. It uses 32 bits Windows Vista Service Pack 2. It was connected to an Asymmetric Digital Subscriber Line (ADSL) SYXEL WiFi Router (10 Mbps uplink and 1 Mbps downlink) with a Fast Gigabit Ethernet Card. In this portable computer we installed a portable version of Apache friends XAMP Server [50]. We only installed a version of XAMP that occupies 46 MB which includes PHP5 and MySQL.

**Figure 18.**
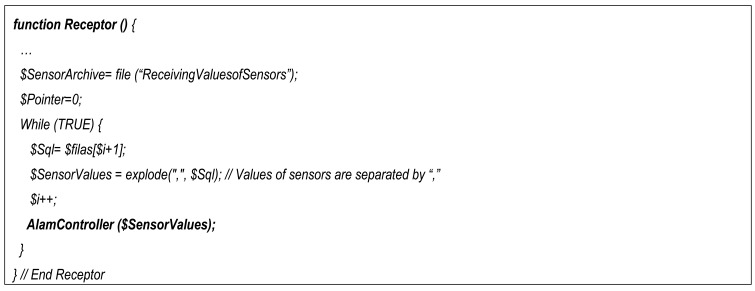
The PHP code of the Receptor.

**Figure 19.**
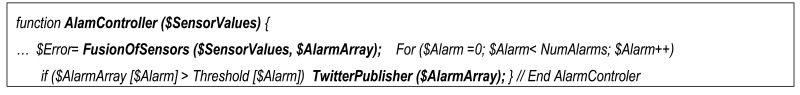
The PHP code of the Alarm controller (including sensors fusion process).

The core PHP code of the Receptor is shown in Figure 18. It basically reads a file in which the sensors' values are stored and pass them to the alarm controller component. In Figure 19 is shown the core PHP code of the Alarm controller (where the fusion of sensors' values is done). In the related literature there are several sensor and multisensory data fusion algorithms [51,52], but in this first step we use an easy data fusion algorithm which is based on a local database which generates the alarm if one of the sensor values is higher or lower than a threshold (although now we are introducing a smart decision and data fusion algorithm as we state in the conclusion section). In order to develop the data fusion system, we connected PHP with a MySQL local database. Queries from PHP allow us to obtain the required data (although this system can be replaced by other systems like Fusion Tables [53]). The array SensorValues containing the values of sensors is passed from the Receptor to the Alarm Controller component. The Alarm Controller makes the fusion of sensor' values returning an array of alarms (AlarmArray) which contains the alarms generated after the fusion of sensor' values. If any of these values if greater than its corresponding threshold then the corresponding calls to Twitter Publisher component will be launched.

**Figure 20.**
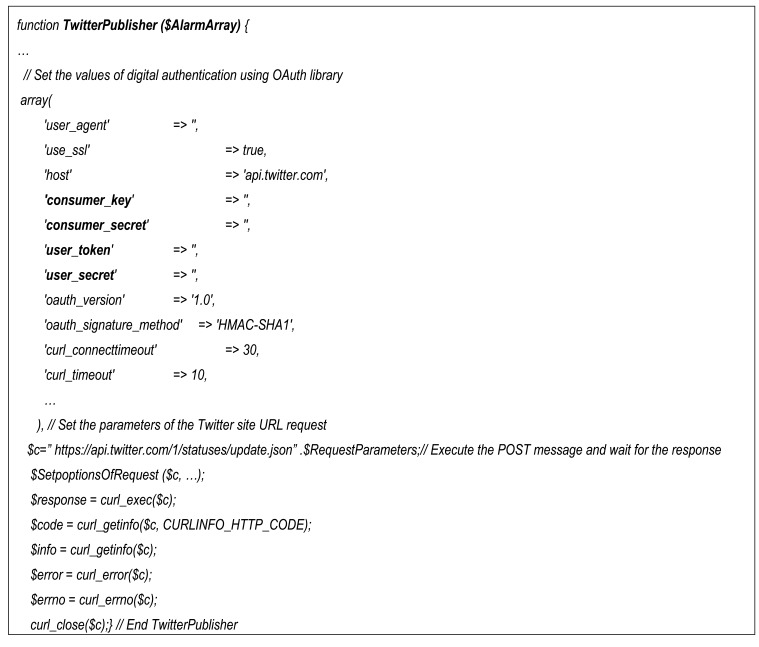
The PHP code of the Twitter publisher.

Finally the core PHP code of the Twitter publisher consists of issuing a POST message that is built with libcurl library [54] and OAuth library for Twitter as is shown in Figure 20. Initially it must set the values of digital authentication using OAuth library filling an array in which the most important parameters are the consumer_key, consumer_secret, user_token, and user_secret. Then it must fill a string with the URL of Twitter site to which it must send the POST message. Finally using the libcurl library the POST transaction is achieved and the results are returned in the $response, $code, $info, $error and $errno variables.

**Figure 21.**
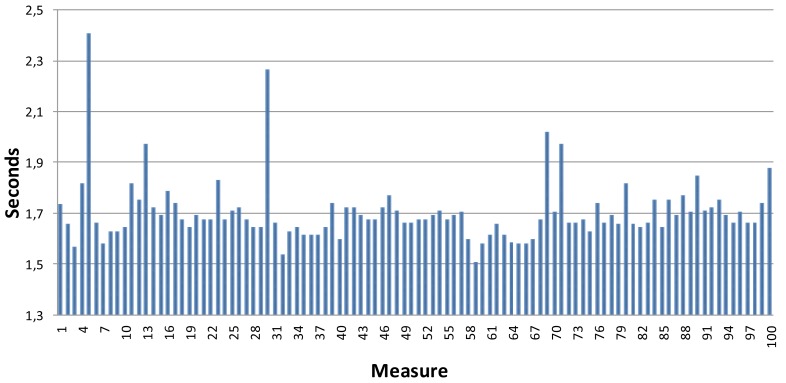
Total time for a very large amount of transactions.

Table 8 shows an example of some measurements obtained after the publication of several and consecutive tweets from the Web server allocated in the Home PC to Twitter.

where x_runtime is the internal time spent by Twitter to process the publication, header size is the size of the HTTP POST message, the request size is the size of the Twitter Web server request message, connect time is the time spent in the connection, and total time is the total amount of time spent in the transaction (let us note that there are other parameters not presented here that influence the values of this parameter). It is clear that the request and response messages are very small and the total time spent in the transaction is also very acceptable. In Figure 21 a graphic with the values of total time for a very large amount of transactions is shown. As shown, the values of the total time are between 1.5 s and 1.8 s (there are a very small set of values that are higher than 2.0 s due to network conditions.

## 6. Conclusions

In this paper we have presented a low-cost smart system that is able to gather data in a home network from electrical household appliances and posts on the Twitter Social Network the appropriate messages according to the parameters sensed from the electrical household appliances. Our assumptions about the existence of one computer at home (named gateway in our proposal), that has Internet access and it can receive the information from the sensors located on each household appliance, has sense nowadays. For that reason we think that our system proposal has an easy deployment with a minor investment at most homes which can be used in any household appliance regardless the model and manufacturer.

The experimental measurements have shown that the overall system performance is good in terms of low delay to publish the tweets in Twitter. Therefore, the notification to the final user is almost instantaneous. Moreover, security in communication and publishing is assured.

We are in the process of improving the system intelligence by the data joining sent by the sensors from the electrical household appliances. The idea is that the gateway be able to take smart decisions and data fusion algorithm according to the information sent by the sensors to avoid publishing false alarms by smart data fusion. Moreover, we are going to include machine learning techniques to provide a QoS-enabled distributed real time system [55]. In this way we can enhance the power of the system.

Although our main future research directions in this work are focused on improving the decision system by adding some intelligence, we are now in the process of adding other devices of the house (such as TVs, alarm clocks, bathroom scales, and even the lights of the house) to the system, which will provide more information to the family. Some of this information may be: the alarm clock in the children room is ringing, there is a light switched on in the house but nobody is at home, *etc.* In order to do this, we must decrease the size of the LSH, so we should work in this direction. Furthermore, we are going to conduct a study in order to see if the behavior of the users changes after using our system for some time.

## Figures and Tables

**Table 1.** Comparative of RS232 sensors.

**Sensor**	**Measures**	**Description**
33X High Accuracy Digital Output Pressure Sensor [32]	Pressure	Pressure Ranges: from 1 up to 1,000 bar
Intelligent Technology and Telecommunications temperature sensor [33]	Temperature	Measures among 0 and 100 °C (LM35) or among −40 and 125 °C (TC1047)
SHT-Rs—Humidity and temperature sensor [34]	Relative Humidity, Temperature	Relative humidity sensor 0 to 100%. Accuracy ±3.5% in humidity measurement Accuracy 0.5% @ 25 °C in temperature measurement
SENSORONE 41X Low Range Digital Output Pressure Sensor Pressure Sensor [35]	Pressure	Pressure Ranges: 10 mbar–300 mbar Accuracy: ±0.1% FS BSL
ALIBABA Indoor temperature sensor. Model Number: CHD301C(V3.0) [36]	Temperature, Humidity	Humidity measure bound: 10%–98%RH (precision: ±3%RH) Temperature measure bound:−10 °C–100 °C (precision: ±0.4 °C)
Airborne Sound Sensor [37]	Sound	Signals up to 100 kHz. Internal microphone.
Kimo instruments multi-function panel meter—CPE 300 [38]	Pressure, Air velocity, Air flow	Measurement of low pressure and\or depression of air or neutral gases from 0/+10 Pa to −1,000/+1,000 Pa
GHM-Messtechnik multi-function meter [39]	Temperature, Air velocity, Relative humidity	0–100%RH, −40–120 °C | GMH 3330
Tenmars electronic multi-function meter: pressure, air velocity and air flow [40]	Pressure, Air velocity, Air flow	0.4–25 m/s | TM-40x
Greisinger multi-function panel meter [41]	Humidity, Temperature, Flow rate measuring	Relative humidity: 0.0 … 100.0%RH Ambient temperature: −40.0 … +120.0 °C Surface temperature: −80.0 … +250.0 °C Flow rate: depending on STS probe
Acmas technocracy (P) LTD: Multiple function weather meter [42]	Anemometer, Humidity meter, Light meter, Thermometer, Sound level meter	Humidity: Max80% RH. Temperature: 0 to 50 °C
Testo 435 multi-functional Measuring Instrument [43]	Anemometer CO_2_ level, Air moisture, Air temperature	Measurements of velocity in the range of 40 to 8,000 fpm (0.2…40 m/s)
Tesco 400 [43]	Temperature, CO, CO_2_, Rpm, mV and mA, % RH, Pressure, Velocity	Humidity probe with 1% accuracy Precision temperature probe with a system accuracy of up to 0.05 °C
Hazmat weather station, P/N 102647 [44]	Wind Speed, Wind direction, Temperature, Relative humidity, Pressure	Operating Temp: −20 to 60 °C Storage Temp: −50 to 70 °C Humidity: 0 to 95%
6 in 1 Digital Multifunction Environment Meter Yh610 [45]	Sound Level, Luminometer, Relative Humidity, Meter, Temperature, Anemometer, Air Flow	Ranges: Temperature: −10–60 °C Relative Humidity: 20%–80%RH Luminometer: 0–50,000 LUM Air Flow: 0.4–20 m/s Meter: 0–999,900 CMM Anemometer: 0–999,900 CFM Sound Level: 30–130 dB
SRF02 Ultrasonic range finder [46]	Sound	15–600 cm
Vibration meter [47]	Vibration meter	Acceleration: 200 m/s^2^ Velocity: 200 mm/s Displacement (p-p): 2 mm

**Table 2.** Notifications of the Refrigerator appliance.

**Alarm ID**	**Title and the Information Processed by Sensors**
1	*Increase the quantity of food on Tray 1 (dairy products)*. Pressure sensor of this tray informs the value is less than 1,500 g. Acoustic sensor is not detecting food closer than 30 cm
2	*Increase the quantity of food on Tray 2 (vegetables)*. Pressure sensor of this tray has informed that the value is less than 400 g. Acoustic sensor is not detecting food closer than 30 cm
3	*Increase the quantity of food on Tray 3 (protein products)*. Pressure sensor of this tray has informed that the value is less than 1,000 g. Acoustic sensor is not detecting food closer than 30 cm
4	*Increase the quantity of drink*. Pressure sensor of this tray has informed that the value is below 1,000 g
5	*Refrigerator Open*. Temperature sensor has informed a temperature above 5 °C on the middle tray

**Table 3.** Notifications of the washer appliance.

**Alarm ID**	**Title and the Information Processed by Sensors**
1	*It is time to hang out my washed clothes*. Vibration sensor informs the washing machine stopped vibrating
2	*The clothes have been washed in 1 hour!* Vibration sensor informs washing machine has been washing during 1 hour. Depending on the washing program the same notification can be launched with a different number of hours
3	*Washing machine does not receive water*. Humidity sensor informs that no water is inside washing machine
4	*Water leaks*. External humidity sensor informs of water leaving the washing machine with no control
5	*Washing machine does not start working*. Vibration sensor informs no vibration is on the machine

**Table 4.** Notifications of the dishwasher.

**Alarm ID**	**Title and the Information Processed by Sensors**
1	*Dishes cleaned!* Vibration sensor detects no vibration and time washing expires
2	*Few dishes inside, nobody eats or drinks in this house!* Pressure and acoustic sensors do not detect dishes nor glasses
3	*Water does not enter or it enters slowly*. Humidity and pressure sensors do not detect low portions of water neither pressure increase
4	*Dishwasher does not dry*. Vibration sensor detects movement but Humidity sensor does not detect drying

**Table 5.** Notifications of the dryer.

**Alarm ID**	**Title and the Information Processed by Sensors**
1	*It is time to put the clothes in the closet*. Vibration sensor informs drying is finished
2	*Too much water in my water container*. Humidity and pressure sensors inform about an irregular process: too much water
3	*The dryer does not heat*. Temperature sensor informs about dryer failures
4	*It is taking too long for the clothes to dry*. Vibration sensor informs that it has been working too much time

**Table 6.** Notifications of the oven.

**Alarm ID**	**Title and the Information Processed by Sensors**
1	*Start to have lunch!* Temperature sensor informs a decreasing temperature inside oven
2	*The meal is being burned!* Pressure sensor detects food inside the oven and temperature sensor informs an increasing temperature inside oven and smoke sensor detects a large amount of smoke
3	*Oven door open too much time*. Temperature sensors detects a low temperature closed to the oven's door and high temperature inside the oven
4	*Fire in the oven!* Temperature sensor detects high temperature inside the oven and the external smoke sensor detects a high concentration of smoke

**Table 7.** LSH device comparison table.

**LSH Device**	**Voltage**	**Average Current**	**Maximum Current**
Xport	5.0 V	0.200 A	0.690 A
Matchport	3.3 V	0.260 A	0.690 A

**Table 8.** Some measurements obtained for different tweets.

**Tweet**	**x_Run Time (s)**	**Header Size (B)**	**Request Size (B)**	**Total Time (s)**	**Connect Time (s)**
1	0.15406	1,461	593	1.731	0.374
2	0.12320	1,461	591	1.653	0.421
3	0.11442	1,464	593	1.56	0.343
4	0.31350	1,461	591	1.81	0.375
5	0.92873	1,461	591	2.403	0.359
6	0.16633	1,461	593	1.654	0.359
7	0.12348	1,461	591	1.575	0.343
8	0.14527	1,461	591	1.622	0.343
9	0.14074	1,461	591	1.623	0.344
10	0.16050	1,461	594	1.638	0.343
